# Bandage contact lens for in-game corneal abrasion can allow immediate return to play

**DOI:** 10.3389/fspor.2024.1351906

**Published:** 2024-03-04

**Authors:** Rohan Kubba, Ganesha R. Kandavel, Joshua Scott, Cesar Roldan, Hayden Jackson

**Affiliations:** ^1^ Department of Ecology and Evolutionary Biology, University of California Los Angeles, Los Angeles, CA, United States; ^2^ Surgical and Medical Ophthalmology, Colvard-Kandavel Eye Center, Encino, CA, United States; ^3^ Department of Sports Medicine, Los Angeles Galaxy Soccer, Carson, CA, United States; ^4^ Department of Ophthalmology, Jules Stein Eye Institute, University of California Los Angeles, Los Angeles, CA, United States; ^5^ Department of Orthopaedics and Sports Medicine, Cedars Sinai Medical Center Kerlan-Jobe Institute, Tarzana, CA, United States

**Keywords:** bandage contact lens, corneal abrasion, immediate return to play, pain management, binocular vision, silicone hydrogel contact lens

## Abstract

While the prevailing treatment for corneal abrasions outside the athletic sphere is the application of a bandage contact lens (BCL), which lessens pain and allows for the maintenance of binocular vision, this is not the case during athletic play. This brief report highlights the advantage of BCLs in treating in-game corneal abrasions, ultimately allowing for an immediate return to play. Additionally, this report summarizes the mechanisms of bandage contact lenses, differentiates them from standard hydrogel contact lenses, and highlights the significant steps necessary to apply the bandage contact lens during an in-game corneal abrasion event. Overall, we link modern ophthalmology clinical practice and sports medicine, allowing for the attenuation of acutely-induced ocular pain to a manageable state.

## Key points

• Bandage Contact Lens can allow an athlete to immediately return to play after suffering a corneal abrasion.
• Bandage Contact Lenses allow for ocular pain management and maintenance of binocular vision, critical for depth perception.

## Introduction

Corneal abrasions have been long recognized as a common ocular injury in sports, occurring in both classical sports like baseball, basketball, and soccer (1) as well as in more recently established sports like pickleball (2). Although protective eye equipment has been recommended to minimize the occurrence of corneal abrasions (3), oftentimes, it is an afterthought to players due to the bulkiness of this equipment potentially affecting gameplay. It is also reasonably likely that if players have a high-power prescription, they will opt for a contact lens to compete in sports instead of goggles as it does not restrict specific fields of vision. Interestingly, while contact lenses are traditionally not considered a part of the protective ocular equipment, studies have shown that the presence of contact lenses may be protective against potential corneal abrasion occurrences, as found in a porcine model (4). Although overuse of contact lenses can promote dryness and lead to corneal abrasions when removing the lens (5), when well lubricated and used in moderation, contact lenses provide a protective shield over the tissue, dispersing potential force over a larger surface area, thereby lowering the likelihood of perforation.

## Deconstructing the Lens

The traditional treatment for a corneal abrasion has been treatment with an eyepatch; however, recent literature suggests that this treatment may be negligible for pain attenuation and insignificant in healing time as compared to non-patching (6). While the usage of standard contact lenses has been revolutionary for sports accessibility to the visually challenged, their usage is not new in sports; however, the usage of bandage contact lenses or (BCLs) would be extensive for the field of sports medicine because of its ability to allow for immediate return to play (IRTP). These are ophthalmologist-prescribed contact lenses primarily marked by having no (Plano) or considerably low prescription power, which contributes to low lens thickness, and concurrently utilize a silicone hydrogel material, which is recognized to have a higher diffusion coefficient of oxygen than standard hydrogel contacts (7). These two factors ultimately culminate in BCLs having a considerably high overall oxygen permeability, which is subsequently crucial to a speedy healing process; however, it is also worth noting that some contact lens manufacturers have recently pivoted to use silicon hydrogel contact lenses for regular contact wearers as well. Interestingly there are varying levels of the water percentage of the lenses themselves across different brands of BCLs (usually from 24% to around 40%) because as the water content of the silicon hydrogel lens increases beyond these thresholds, their oxygen permeability tends to decrease (8). Moreover, a variety of silicone hydrogel materials themselves are used for bandage contact lenses across different companies, and currently, these are senofilcon A, lotrifilcon A, and balafilcon A. Interestingly, a 2014 study found that for pain management following photorefractive keratectomy surgery, patients primarily preferred using a senofilcon A-based BCL, then ranked a lotrifilcon A-based BCL second, followed thirdly by a balafilcon A-based BCL (9). An example BCL is the Air Optix Night and Day Aqua Contact Lens from Alcon, which is made with lotrifilcon A and is manufactured in Indonesia.

Albeit Return to Play (RTP) has its own set of conditions, as explained by Acar et al. (2020) (10), using a bandage contact lens in the case of an in-game corneal abrasion can allow for noticeable pain relief and retention of binocular vision, which is critical for depth perception. Although the maintenance of binocular vision is helpful to the player, especially in sports involving a ball, the intended value of the BCL is in its ability to immediately decrease ocular pain and accelerate re-epithelialization in the cornea. While this proposal is seemingly ambitious, this was the reality for a player on the LA Galaxy in 2021 as he reportedly developed a corneal abrasion and returned to the game after team trainer Cesar Roldan administered a bandage contact lens, following the steps in the following paragraph elucidated by LA Galaxy team ophthalmologist Dr. Rom Kandavel.

## Application Process

Research has shown that BCLs can quickly alleviate excruciating ocular pain and provide binocular vision after the onset of a corneal abrasion (11), and BCLs are now a preferred treatment option for many other ocular complications and surgeries including postoperative Muller's Muscle-Conjunctival Resection (12). Mechanistically, the bandage contact lens protects against friction of the eyelid and air and provides a structure for re-epithelialization and, therefore, healing. While bandage contact lenses protect against stromal ulceration, wearing them does increase the risk of developing, among others, corneal edema and vascularization peripherally. However, this can be minimized with the usage of preventative measures. In general, only players with a glancing blow or direct “poke” to the eye should be considered for this BCL intra-game intervention. The presence of double vision, lid laceration, or periorbital ecchymosis may hint at a more significant injury and, therefore, the athlete should not return to play.

Before administering the bandage contact lens, the player should be given proparacaine to anesthetize the eye, followed by drops of a prophylactic antibiotic to treat the abrasion, specifically 4th generation drugs like fluoroquinolone and Moxifloxacin and then followed by a nonsteroidal anti-inflammatory eye drop such as Bromfenac (13) to reduce inflammation and further attenuate pain. If the player regains good vision in the affected eye with the BCL in place, this would indicate that the abrasion is not centrally located and the BCL is therefore effective in restoring usable vision. If vision has not been restored, a decision on RTP should be made based on the impaired visual result by the team trainer/coaching staff. Following the end of the game, the player should immediately be clinically evaluated by the team ophthalmologist to ensure the wound is healing properly, there is no foreign body, and the athlete is not in unreasonable discomfort. The BCL should be left in place with the same fluoroquinolone eye drop used four times daily until the ophthalmologist has been consulted.

## Discussion

**Figure 1:**
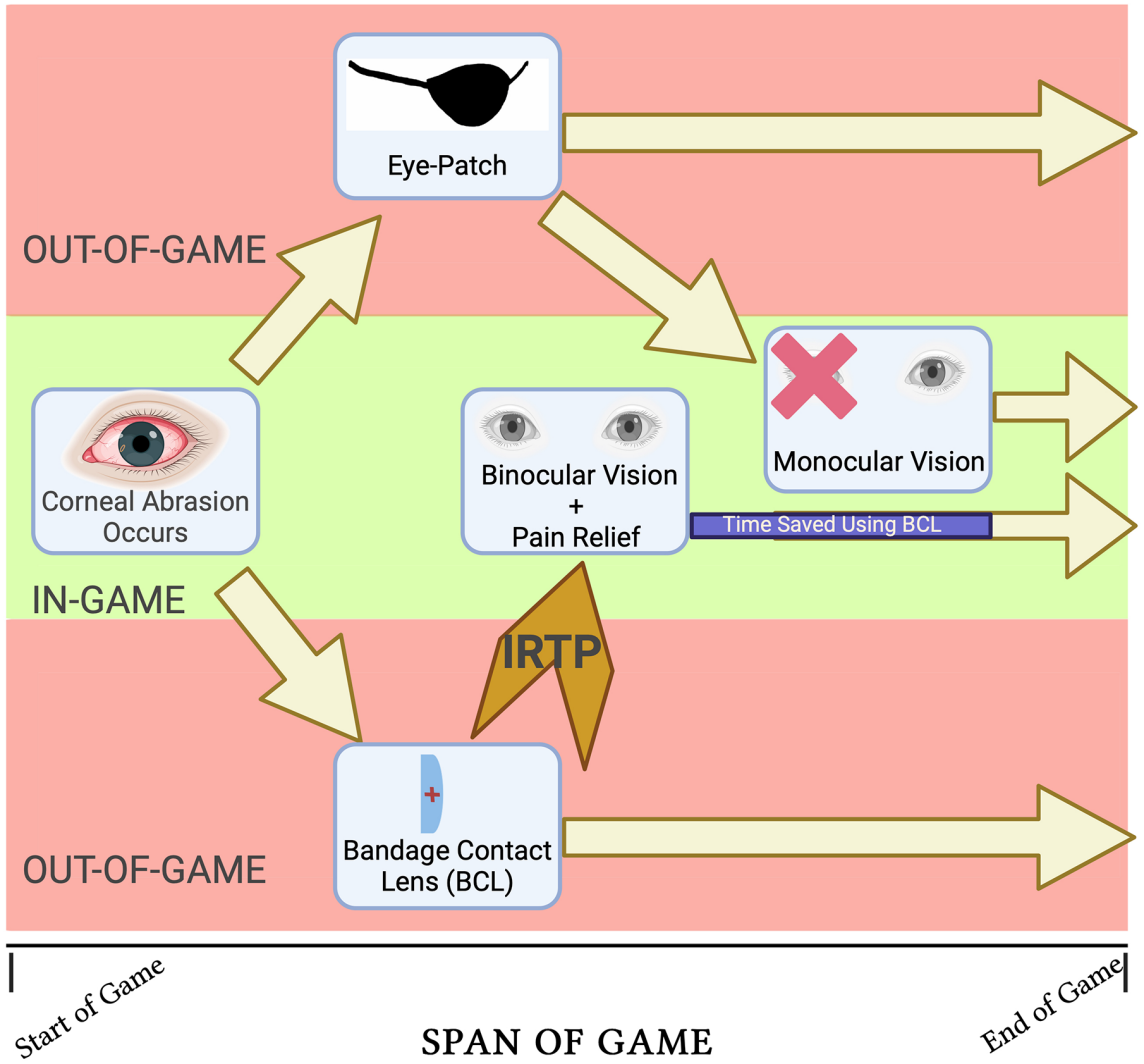
Opportunity cost schematic of employing a BCL vs eye-patching for in-game corneal abrasion.

Ultimately, the team ophthalmologist must critically decide whether the athlete qualifies for IRTP based on whether the BCL reasonably alleviates pain and allows for binocular vision. Figure 1 summarizes the temporal benefit of using a bandage contact lens during an in-game corneal abrasion vs. patching. Regardless of whether the patient is an athlete or not, the BCL is quickly becoming the preferred treatment option for corneal abrasions, and while BCLs are conventional knowledge to most ophthalmologists, their influence in sports medicine for immediate return to play will certainly be a game-changer.

## Data Availability

The original contributions presented in the study are included in the article/Supplementary Material, further inquiries can be directed to the corresponding author.
